# A Comparative Evaluation of Effect of Different Chemical Solvents on the Shear Bond Strength of Glass Fiber reinforced Post to Core Material

**DOI:** 10.5005/jp-journals-10005-1263

**Published:** 2015-02-09

**Authors:** Ashish Sharma, Firoza Samadi, JN Jaiswal, Sonali Saha

**Affiliations:** Postgraduate, Department of Pedodontics and Preventive Dentistry, Sardar Patel Postgraduate Institute of Dental and Medical Sciences Lucknow, Uttar Pradesh, India; Profesor and Head, Department of Pedodontics and Preventive Dentistry, Sardar Patel Postgraduate Institute of Dental and Medical Sciences Lucknow, Uttar Pradesh, India; Professor and Director, Department of Pedodontics and Preventive Dentistry, Sardar Patel Postgraduate Institute of Dental and Medical Sciences Lucknow, Uttar Pradesh, India; Senior Lecturer, Department of Pedodontics and Preventive Dentistry, Sardar Patel Postgraduate Institute of Dental and Medical Sciences Lucknow, Uttar Pradesh, India

**Keywords:** Shear bond strength, Glass fiber post, Core build up, Composite resin, Silane coupling agent, 6% Hydrogen peroxide, 37% Phosphoric acid.

## Abstract

**Aim:** To compare the effect of different chemical solvents on glass fiber reinforced posts and to study the effect of these solvents on the shear bond strength of glass fiber reinforced post to core material.

**Materials and methods:** This study was conducted to evaluate the effect of three chemical solvents, i.e. silane coupling agent, 6% H_2_O_2_ and 37% phosphoric acid on the shear bond strength of glass fiber post to a composite resin restorative material. The changes in post surface characteristics after different treatments were also observed, using scanning electron microscopy (SEM) and shear bond strength was analyzed using universal testing machine (UTM).

**Results:** Surface treatment with hydrogen peroxide had greatest impact on the post surface followed by 37% phosphoric acid and silane. On evaluation of the shear bond strength, 6% H_2_O_2_ exhibited the maximum shear bond strength followed in descending order by 37% phosphoric acid and silane respectively.

**Conclusion:** The surface treatment of glass fiber post enhances the adhesion between the post and composite resin which is used as core material. Failure of a fiber post and composite resin core often occurs at the junction between the two materials. This failure process requires better characterization.

**How to cite this article:** Sharma A, Samadi F, Jaiswal JN, Saha S. A Comparative Evaluation of Effect of Different Chemical Solvents on the Shear Bond Strength of Glass Fiber Reinforced Post to Core Material. Int J Clin Pediatr Dent 2014;7(3):192-196.

## INTRODUCTION

Endodontically treated teeth with significant loss of coronal tooth structure may require placement of post to ensure adequate retention of a core foundation. Fiber posts together with composite core build-up materials are currently perceived as promising alternatives, for providing more esthetic outcomes.^1^ Limited studies have been conducted to obtain an ideal surface area of these fiber posts so as to enhance its adhesion to core material. Hence, the present study has been taken up to evaluate effect of newer chemical solvents, i.e. 6% hydrogen peroxide (H_2_O_2_) and 37% phosphoric acid on shear bond strength of glass fiber posts to core material.

## MATERIALS AND METHODS

The present *in vitro* study was carried out in the Department of Pedodontics and Preventive Dentistry, Sardar Patel Postgraduate Institute of Dental and Medical Sciences, Lucknow in collaboration with Central Institute of Plastic Engineering and Technology (CIPET), Lucknow and Birbal Sahni Institute of Palaeobotany (BSIP), Lucknow. This study was conducted to evaluate the effect of three chemical solvents, i.e. silane coupling agent, 6% H_2_O_2_ and 37% phosphoric acid on the shear bond strength of glass fiber post to a composite resin restorative material. The changes in post surface characteristics after different treatments were also observed, using scanning electron microscopy (SEM).

### Preparation of Samples

**Fig. 1:**
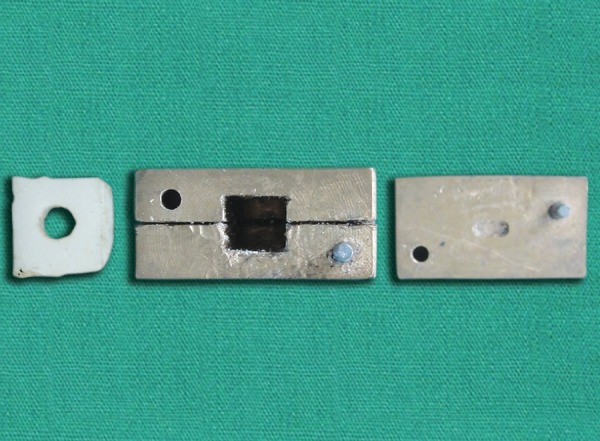
Custom made brass mold

**Fig. 2:**
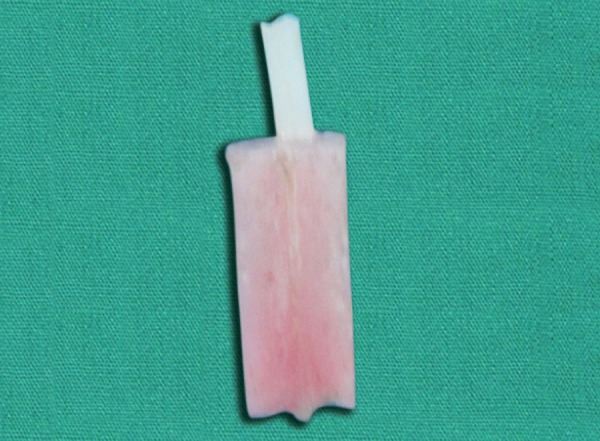
Glass fiber post pieces were embedded horizontally in the center

Fifteen posts were cut into two pieces of 4 mm length and 0.1 mm thickness using a diamond rotary instrument and then placed in a slot of the head of a custom made brass mold (Fig. 1). Adequate amount of separating media was applied between the two halves of the mold to ensure easy removal of the resin block. A small amount of autopolymerising resin was mixed in a dap-pen dish and flled into the mold. The head of the mold with the glass fiber post piece was placed into position and pressed with a custom made clamp. Glass fiber post pieces were embedded horizontally in the center (Fig. 2). The exposed portions of these posts were successively ground until fattened fush with the acrylic resin, to create a standard smooth surface. The specimens thus prepared were ultrasonically cleaned with distilled water for 2 minutes and dried with compressed air.

*Division of samples and surface treatment*: The specimens were divided into 3 groups of 10 specimens each, depending on the surface treatment used: Group I (Control Group) – Silanization of the post surface with a brush soaked in a silane coupling agent for 60 seconds, according to the manufacturer's recommendation. Group II: Etching with a cotton pellet soaked in 6% H_2_O_2_ for 20 minutes at room temperature. Group III: Etching with a cotton pellet soaked in 37% phosphoric acid for 20 seconds at room temperature. The treatment times of H_2_O_2_ and 37% phosphoric acid were determined according to previous studies.^2-4^

**Fig. 3:**
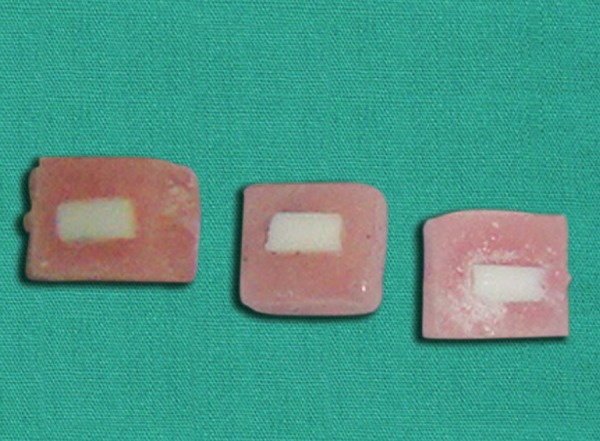
Specimens

*Core build up procedure*: A white polytetrafuoroethy-lene mold, 8 × 6 mm thick, with 1.5 × 1.5 mm hole in the center was used to add composite resin to the treated specimen surfaces. Composite resin was polymerized for 20 seconds. The specimens were then stored in water for 24 hours at room temperature (Fig. 3).

**Fig. 4:**
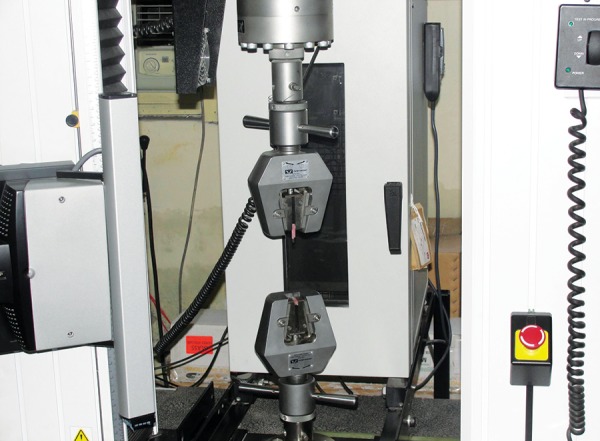
Universal testing machine

**Fig. 5:**
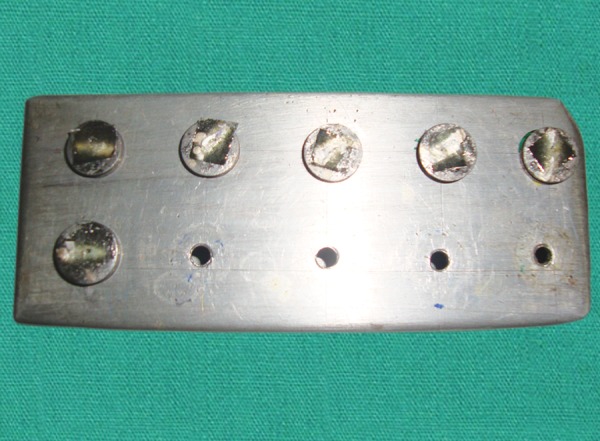
Specimens coated with gold using a sputter coater before SEM analysis

*Laboratory procedure*: The specimens were then mounted in a custom fixture for determination of shear bond strength. Universal testing machine (UTM) (Fig. 4) equipped with a sharp blade was used to deliver the shearing force with the shearing blade perpendicular and directed at interface of fiber post and composite core. Shear bond strength of each composite resin specimen was measured in terms of MPa, F/A (force per unit area). To evaluate the effect of the chemical treatments on the surface of the post material, additional glass fiber posts without core material were used . The surfaces of the posts were treated in the same manner as described above. All specimens were coated with gold using a sputter coater (Polaron SC 7640, VG Microtech, UK) (Fig. 5) and examined under a feld emission SEM (LEO 430, LEO Electron Microscopy Ltd, Cambridge, UK) at 10 kV. The magnified (250×) SEM photomicrographs were evaluated visually.

## STATISTICAL ANALYSIS

Statistical analysis was performed using the SPSS 15 statistical software version. The analysis of variance (ANOVA) and post hoc tests (Tukey-HSD) were performed to know the effect of each variable and to reveal the statistical significance.

## RESULTS

Table 1 shows mean shear bond strength in different study groups.

**Fig. 6:**
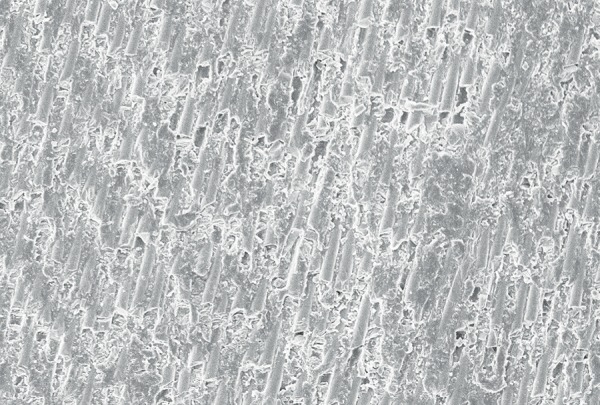
The SEM image of fiber post, after treatment with silane (control group) which revealed less exposed fibers after treatment

**Fig. 7:**
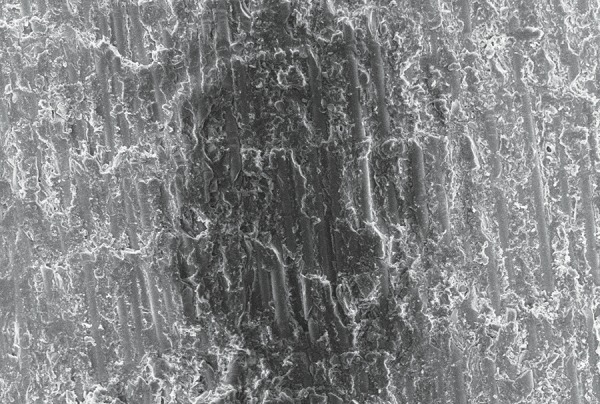
The SEM image of fiber post, after treatment with 6% H_2_O_2 _(group II) revealing more exposed fibers after treatment

**Fig. 8:**
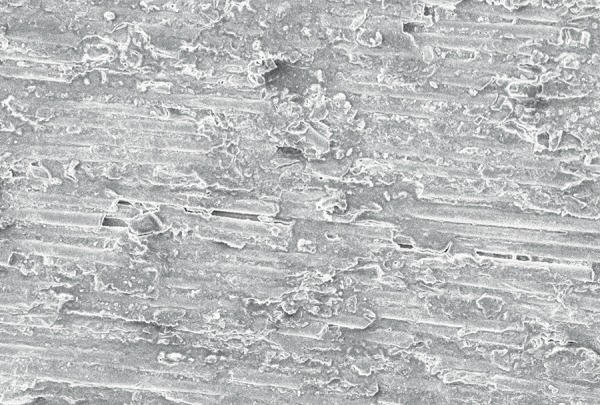
The SEM image of fiber post, after treatment with 37% phosphoric acid (group III) revealing more exposed fibers compared to group I (control group) but less exposed fibers in comparison to group II (6% H_2_O_2_)

**Table 1:** **Table 1:** Shear bond strength (MPa) in different groups

*Sl. no.*		*Groups*		*No. of samples*		*Mean*		*SD*		*Minimum*		*Maximum*	
1.		I		10		19.41		0.34		19.01		19.96	
2.		II		10		25.52		0.35		24.86		25.88	
3.		III		10		21.14		0.48		20.36		21.74	

SD: Standard deviation

On evaluation of the shear bond strength, group II (6% H_2_O_2_) exhibited the maximum shear bond strength followed in descending order by group III (37% phosphoric acid) and group I respectively. The changes in post surface after different chemical treatments were observed by SEM evaluation which revealed that the post surface morphology was modified and surface treatments dissolved the epoxy resin matrix and exposing the quartz and glass fibers in the posts. Figure 6 shows the SEM image of fiber post, after treatment with silane (Control Group) which revealed less exposed fibers after treatment. Figure 7 shows the Scanning Electronic Microscopic image of fiber post, after treatment with 6% H_2_O_2_ (Group II) revealing more exposed fibers after treatment. Figure 8 shows the SEM image of fiber post, after treatment with 37% phosphoric acid (Group III) revealing more exposed fibers compared to group I (control group) but less exposed fibers in comparison to group II (6% H_2_O_2_).

## DISCUSSION

Endodontically treated anterior teeth are often severely damaged by decay, excessive wear or previous restorations, resulting in a lack of coronal tooth structure.^2^ The post and core system is considered the most appropriate restoration for such teeth and the primary purpose of a post is to retain a core in a tooth with extensive loss of coronal tooth structure. With changing trends, in comparison to metallic post, nonmetallic materials are used for the fabrication of posts based on the carbon-fiber reinforcement principle.^3^ Studies by Duret et al (1990) have also shown that these posts have a high tensile strength and modulus of elasticity, similar to dentin. Fiber-posts have an additional advantage of fexing under load and as a result distributing stresses between the post and the dentin. Currently available fiber-based posts are essentially composite materials composed of fibers of carbon or silica surrounded by a matrix of polymer resin, usually an epoxy resin.^5,6^ To enhance the retention of a post, core build up is done so as to increase the surface area. Composite is used as a core material for fiber post. The clinical success of a post-and-core restoration depends on the composite resin selected and the quality of the post and core interface, where materials of different compositions are in intimate retentive contact.^7^ Monticelli et al (2005) investigated different procedures for improving the interfacial bond strength between posts and resin-based materials using chemical and mechanical surface treatments. These procedures may be divided into 3 categories: silanization and/or adhesive application like acid etching, airborne-particle abrasion, and silica coating; and treatments that combine both a micromechanical and a chemical component.^5^

Baskin et al (1979) inferred that the rationale for conditioning the fiber post relies on the purpose of removing a surface layer of epoxy resin, rendering more quartz fibers available for silanization, and improving the fiber post surface bonding area.^8^ The use of silane coupling agents improves surface wettability and creates a chemical union between the glass fibers and resin-based materials.^9^ The methacryloxyl group in this silane coupling agent probably enables a chemical union to be achieved between the silane-treated fibers post and the methacrylate-based resin composite matrix, thereby improving interfacial strength. The beneft of applying silane-coupling agents as adhesion promoters has also been reported by Aksorn-muang et al (2004) and Goracci et al (2005).^10^ However, the post/composite joint still remains relatively weak. In an effort to enhance the bond strength, i.e. the chemical union between the fiber post and core material, newer chemical solvents along with silane are being tried to obtain better retention of the endodontically treated tooth which were restored by fiber post using fowable composite as core material. The chemical solvents used in earlier studies were H_2_O_2_, 5% hydrofuoric acid, 10% hydrofluoric acid, 21% sodium ethoxide, potassium permanganate and methylene chloride.^11^ Though, it has been reported that 37% phosphoric acid has been used for etching procedures but further research has to be done to observe the effect of 37% phosphoric acid on the interfacial strength between fiber post and composite core material. While taking into consideration the chemical solvents used in previous studies, H_2_O_2_ is the most appropriate chemical solvents as it is a better oxidizing agent when compared to others. By removing a surface layer of epoxy resin, etching with H_2_O_2_ created a larger surface area of exposed quartz fibers available for silaniza-tion. The spaces between these fibers provide additional sites for micromechanical retention of the fowable resin composites.^12^ Etching fiber posts with hydrofuoric acid, however considerably affected the integrity of the fiber posts. On the contrary, H_2_O_2_ etching is a considerably milder technique with the exposed quartz fibers remaining smooth and leaving the underlying epoxy resin matrix intact after the etching procedures. Insufficient bond strength is the main failure reasons in composite resin restorations even when used as a core material. However, using the so-called acid etch technique and bonding systems leads to a significant reduction in microleakage and provide the surface with the most retentive appearance. In the advancement of dental materials 6% H_2_O_2_ and 37% phosphoric acid were considered as highly effective chemical solvents in terms of its application to fiber post along with silane.^13^ In this study, a comparison has been made between these two chemical solvents. As there has been no previous study conducted comparing both these chemical solvents together, the present study had been taken up. The outcome of these chemical solvents on the surface of the post had been analyzed using SEM and its effect on the adhesion between the fiber post and the core material has been checked using UTM by calculating the shear bond strength of post and core. SEM evaluation showed dissolution of the epoxy resin matrix resulting in exposed quartz fiber components of the post and creation of ‘retention spaces’ among the fiber that appeared to be completely infltrated by the core materials.^14^ The SEM study revealed more exposed fibers after treatment with 37% phosphoric acid when compared to group I (control group) but less exposed fibers in comparison to group II (6% H_2_O_2_). According to Yenisey et al (2008) and Monticelli et al (2005). SEM images are considered as the most appropriate tool in ‘*in vitro*’ study as they provide a detailed description of the effect of chemical solvents.^2,5^ To measure the effect of chemical solvents on the adhesion between post and core material in a quantitative way, the shear bond strength between fiber post and composite resin was evaluated using UTM showing that chemical treatment with 6% hydrogen peroxide exhibited maximum shear bond strength followed in decreasing order by 37% phosphoric acid and silane respectively.^15^ In accordance to the present study, similar results were also found by Yenisey et al (2008), Monticelli et al (2005) and Vano et al (2006).^2,5,16^ Vano et al (2006) stated that may be due to the formation of a multilayer structure that resulted in reduction of the effectiveness of silane coupling, as the number of free methacrylate groups were reduced, and hence cohesive failure may occur within the silane coating. Hence, the low bond strength values obtained for silane may be due to the absence of free radicals in the pre-polymerized post that is performed under heat and vacuum by the manufacturer. As an oxygen inhibition layer is absent, the bonding is poor.^16^ Results of the present study also revealed maximum shear bond strength in 6% H_2_O_2_ followed in descending order by group III (phosphoric acid) which is in accordance to the study by Monticelli et al (2005), who stated that the use of H_2_O_2_ pre-treatment and silane application significantly enhanced the interfacial structure between fiber posts and core materials.^5^ According to Monticelli et al (2008) H_2_O_2_ etching provides an easy, effective and clinically feasible method for enhancement of interfacial strengths without the need of employing extremely corrosive liquids in a clinical setting. It acts by removing a surface layer of epoxy resin and hence larger surface areas of exposed quartz fibers are available for silanization. The spaces between these fibers provide additional sites for micromechanical retention of resin composites.^13^ Ferrari et al (2000) conducted a microscopic study to compare H_2_O_2_ and hydrogen fuoride for bonding fiber post into root canal and concluded that H_2_O_2_ etching was a milder technique with the exposed quartz fibers remaining smooth and leaving the underlying epoxy resin intact after the etching procedures.^17^ Vano et al (2006) evaluated the infuence of hydrogen peroxide, hydrofuoric acid, silanization, and a bonding agent on the microtensile bond strength of glass fiber posts to different composite resins. The authors concluded that treatment of glass fiber posts with hydrogen peroxide was an effective method that can improve the clinical performance of methacrylate resin based glass fiber posts.^16^ In spite of many chemicals used for etching no earlier studies have been of done to evaluate the effect of 37% phosphoric acid post surface on fiber posts. Hence, the present study has been taken up to evaluate the infuence of Phosphoric acid on micro-tensile bond strength of glass fiber posts to different composite resin. According to Yassin et al (2005), etching with 37% phosphoric acid resulted in significant and reliable bond strength and Yamada et al (2002) observed that phosphoric acid conditioning generated a more durable and higher bond strength.^18^
